# Willingness to pay for solid waste management services and associated factors in Mbarara District, Southwestern Uganda

**DOI:** 10.1371/journal.pgph.0005175

**Published:** 2026-03-26

**Authors:** Erastus Tugume, Abraham Muhwezi, Tom Murungi, Julius Kyomya, Edgar Mulogo Mugema, Richard Migisha, Moses Ntaro

**Affiliations:** 1 Department of Community Health, Faculty of Medicine, Mbarara University of Science and Technology, Mbarara, Uganda; 2 Department of Pharmacy, Faculty of Medicine, Mbarara University of Science and Technology, Mbarara, Uganda; 3 Uganda Public Health Fellowship Program, Uganda National Institute of Public Health, Kampala, Uganda; 4 Department of Physiology, Mbarara University of Science and Technology, Mbarara, Uganda; PLOS: Public Library of Science, UNITED STATES OF AMERICA

## Abstract

Willingness to pay (WTP) for solid waste management services is essential for sustainability, yet it remains unassessed in Mbarara District. This study assessed the prevalence of WTP for solid waste management services and the associated factors among households in Mbarara District, Southwestern Uganda.This was a quantitative cross-sectional survey conducted among 250 individuals in households of Bwizibwera-Rutooma and Rubindi-Ruhumba town councils, Mbarara district. We used multistage sampling to select the administrative units and consecutively selected the participants from households. Data were collected using a 26-item interviewer-administered questionnaire, entered in Microsoft Excel and transferred to STATA version 17.0 for cleaning and analysis. Continuous variables were summarized using means and standard deviations, while categorical variables were presented as frequencies and proportions. Bivariate and multivariable logistic regression analysis at a 95% level of confidence was done to identify factors associated with WTP for solid waste management services.Overall, 62% (156/250; 95% C.I.: 56.2%-68.2%) of the participants were willing to pay for solid waste management services. The majority, 64.1% (100/156), were willing to pay one thousand Uganda shillings or more for SWM. Factors associated with WTP for solid waste management services were; being male (aOR = 2.4, 95% CI: 1.2-4.6; *p*-value = 0.011), having a monthly income>28 USD (aOR = 2.2, 95% CI: 1.7-4.1; *p*-value = 0.015), disposing wastes using town council services (aOR = 7.75, 95% CI: 1.35-44.47; *p*-value = 0.022) and receiving weekly waste collection services (aOR = 2.62; 95% CI: 1.06-6.50; *p* = 0.038). The WTP for solid waste management services was relatively high and positively associated with being male, having a monthly income of>100,000 UGX, reliance on town council collection services, and weekly waste collection. Policymakers and local authorities should prioritize affordable, reliable waste collection and inclusive measures, like subsidies and targeted community engagement to boost participation and sustain waste management.

## Introduction

Global solid waste generation has increased significantly in recent decades due to urbanization and rising consumption [1]. For example, cities worldwide produced an estimated 1.3 billion tons of municipal solid waste in 2012 [2], and solid waste generation in towns is expected to surge by 70% by the year 2050 [1,3]. This surge in waste has exceeded the capacity of many waste management systems, especially in low- and middle-income countries. In numerous developing cities, waste services already utilize 20–50% of municipal budgets, yet they often collect only a portion of the waste produced [4,5]. About 125 million tons of solid waste are generated annually across Africa, with sub-Saharan countries accounting for roughly 65% of that total, despite low collection rates [6]. Uganda generates about 0.55 kg/capita/ day, with the collection rates being below 50% [7,8].

The rapid urbanization and growth of cities and towns in Uganda [9] have exacerbated solid waste generation, posing significant challenges to effective solid waste management [8]. This has overwhelmed the capacities of the municipal councils to manage the large quantities of waste generated [10]. These municipal councils are allocated limited funds and resources to sustain waste collection services [11]. As a result, there has been an increase in poor solid waste management practices posing significant health risks and environmental degradation [12,13]. As a result, privatizing solid waste collection has been adopted to address poor solid waste management practices and alleviate the challenges faced by the public sector during waste collection [14–16].

Willingness to pay (WTP) for solid waste management services is a critical indicator of community support for sustainable waste systems, especially in low-resource settings. Studies across sub-Saharan Africa show considerable variation in WTP levels, with higher prevalence observed in Nigeria (64.4%) [17], Ethiopia (83.5%) [18] and Tanzania (63%) [19]. However, studies in Uganda have reported WTP for solid waste management to be 48% in Lira [20] and 64% in Kawempe division, Kampala [21]. The WTP levels have reportedly been influenced by factors such as monthly income, gender, education, household size, amount of waste generated, distance to collection sites and current service quality [17,18,20–23].

With the elevation of several town councils in Mbarara District in 2019, there has been an influx of people seeking economic and social opportunities in these growing urban centres [10]. This population growth, however, has brought significant challenges, including increased solid waste generation [10,16,24]. The district produces about 31.4 tons of garbage daily, of which the town councils of Bwizibwera-Rutooma and Rubindi-Ruhumba alone contribute approximately 8.0 tons per day [24,25]. Yet, due to limited financial and logistical capacity, the councils are only able to collect about 60% of this waste [10,16]. Furthermore, municipal reports indicate that fewer than 30% of residents in these town councils (Rubindi-Ruhumba and Bwizibwera-Rutooma) currently pay fees for waste collection services.

Assessing willingness to pay is essential because it supports sustainable, cost-recovery models in contexts where public funding is limited. Unlike previous studies in Uganda conducted in long established urban settings such as Lira [20] and Kawempe [21], this study focuses on the newly urbanized town councils, where service delivery modalities, fee enforcement mechanisms, and household payment mechanisms are still evolving. By examining WTP in relation to the existing mix of private and public waste collection services, this study provides policy relevant evidence to inform cost-recovery and service expansion strategies in emerging urban local governments. Therefore, we assessed households’ willingness to pay for solid waste management and associated factors in Mbarara district, Southwestern Uganda.

## Materials and methods

### Study design

This was a cross-sectional Analytical study conducted in households of Rubindi-Ruhumba and Bwizibwera-Rutooma town councils within Mbarara district, Southwestern Uganda between 11^th^ March 2025–14^th^ April 2025. Mbarara District is located in south-western Uganda and comprises one county, Kashari County, which is divided into two constituencies: Kashari North and Kashari South. The district lies within latitude 0°28’00.0” S (0.4666700°) and longitude 30°34’60.0” E (30.58333300°). Kashari County is composed of six sub-counties (SC) and five Town Councils (TC): Bubaare SC, Bukiro SC, Kagongi SC, Kashare SC, Rubaya SC, Rubindi SC, Nyabisirira Tc, Rubindi- Ruhumba TC, Bwizibwera- Rutooma Tc, Rwanyamahembe TC and Bukiro TC (S1 Fig). The district has an estimated population of 257,222 people, of whom 48.3% are male, and 51.7% are female, with the majority being of the Banyankole ethnic group. The district is located 270km away from Kampala, the capital city of Uganda. In Mbarara district, more than 30% of the waste produced daily (over 200 tons) is left uncollected, improperly processed, or disposed of in unsuitable locations [24,25]. This is mainly due to limited funding to municipal councils to facilitate waste management services, as reported in the literature [24,25]. This has raised sanitation and health concerns in the area, as poor waste disposal-related diseases have increased in the population [26,27].

### Study population and eligibility criteria

The study population comprised adults aged (≥18 years) residing in households within Rubindi–Ruhumba and Bwizibwera–Rutooma Town Councils in Mbarara District. Only individuals who had lived in these areas for at least six months were considered part of the target population, ensuring adequate familiarity with local waste management practices and community dynamics.

### Inclusion criteria

Participants were eligible for inclusion if they were adults (≥18 years), permanent residents of the selected town councils, and had lived in the area for a minimum of six months. Individuals also needed to be capable of understanding the study procedures and providing informed consent.

### Exclusion criteria

We excluded individuals if they had cognitive impairments or serious illnesses that limited their ability to meaningfully participate in the interview process. Anyone unable to provide informed consent due to health-related or functional limitations was also excluded from the study.

### Sampling procedure and sample size

Out of 5 Town councils in Mbarara district, 2 Town councils were randomly selected, 2 wards per Town council were chosen using probability proportional to size sampling, and 4 villages were selected in each town council. In each village, we selected the first household by lottery and then used consecutive sampling to choose the remaining households. Approximately 40 households were picked from each village in Rubindi-Ruhumba, while about 20 households were selected from each village in Bwizibwera-Rutooma town council. Within each selected household, we followed the eligibility criteria (household heads aged 18 years and older and residents for at least 6 months). Only one participant was selected from each household in case more than one of them was eligible.

The sample size was determined using Krejcie and Morgan’s (1970) method, with a Z-value of 1.96 corresponding to a 95% confidence level, a margin of error of ±5%, and an anticipated response proportion of 80%. Based on a total population of 23,800, the minimum required sample size was calculated as 240 participants. A total of 250 eligible participants were included in the analysis, which provided slightly higher statistical power to detect meaningful differences between study variables. Proportional allocation was used according to the population distribution of the two town councils: Bwizibwera-Rutooma (32%) contributed 77 participants, while Rubindi-Ruhumba (68%) contributed 163 participants.

### Data collection tool, methods and procedures

The study used a researcher-administered questionnaire developed from similar previous studies to collect data [21]. Relevant items were reviewed and selected based on their alignment with the study objectives and local context. Some questions were modified for clarity and contextual relevance, and additional items on household waste practices and willingness to pay were developed by the research team. The tool was further reviewed by an Environmental Health Expert (M.N) with a PhD in Environmental Health, who assessed its contents, relevance, clarity, and technicality regarding willingness to pay. The expert ensured that the items adequately captured key environmental health constructs, were scientifically sound, and were appropriate for the local setting. Feedback provided was incorporated to refine the instrument before data collection The tool had 26 items that were organized under 3 main categories namely: sociodemographic variables, household waste characteristics, and frequency of collection by service providers. The tool also collected data on outcome variable which was willingness to pay. The tool was pretested and refined to improve clarity and flow before use in the main study. The adaptation focused on ensuring contextual relevance, clarity of wording, and alignment with local waste management practices.

Data were collected by trained research assistants who were fluent in the local language (Runyankore), enabling them to effectively support and accommodate participants who were not proficient in English. The tool consisted of the sociodemographic characteristics (age, sex, education level, marital status), willingness to pay for solid waste management, and factors associated with willingness to pay for solid waste management. Data was collected using researcher-administered questionnaires with participants in their households. Each participant was screened for eligibility, consented and recruited into the study. Participants were interviewed in private spaces of their choice. Each interview took approximately 15–20 minutes, and each study tool was checked for completeness after every interview.

The interviews were conducted by three trained research assistants who had a Bachelor of Science in Public Health. Research assistants selected the first household across the road and selected the rest of the households consecutively in each village. In addition, field supervisors monitored the data collection process, conducted spot checks to verify adherence to study procedures, and reviewed completed questionnaires for completeness and accuracy. Where necessary, re-interviews were conducted to confirm responses and resolve any inconsistencies.

### Study variables

The dependent variable in this study was willingness to pay for solid waste management, which was measured using dichotomous responses (willing to pay/not willing to pay). The outcome question was, “Are you willing to pay for the management of Solid waste services in this Town?” The follow-up questions evaluated the amount individuals would be willing to pay (<500 UGX, 500–1,000 UGX, and >1,000 UGX) and the frequency of collection (Daily, Weekly, Monthly, and not regularly collected). On the other hand, independent variables included: sociodemographic variables (including sex, age, education level, marital status, house ownership, and monthly income). Others were household waste characteristics (including quantity of waste generated, places of waste disposal, and frequency of collection by service providers). Quantity was measured using the number of polythene bags filled per week. For this study, a polythene bag was defined as the standard black garbage bag commonly used in households for waste storage, which typically holds approximately 3–5 kg of mixed household waste. And the outcome variable is willingness to pay.

### Quality control

To ensure accuracy, consistency, and appropriateness of the study tool, several quality control measures were undertaken before data collection. The study tool was pretested among 20 individuals in Ibanda district, and the information obtained was used for adjusting the tool before data collection. The purpose of the pretest was to evaluate question comprehension, flow, and timing. All data collectors were trained on the study purpose, ethical and data collection procedures. Feedback from the pretest were incorporated into the final instrument, improving its clarity.

### Data management and analysis

All digital files were stored encrypted, password-protected and physical records were stored in locked cabinets, only accessible to the research team. A data entry screen was created in Microsoft Excel 2016, and data was entered in duplicates by research assistants. Data cleaning was done, and the data were exported to STATA version 17.0 for analysis.

Continuous Variables were summarized using means and standard deviation, while categorical variables were analyzed using proportions and frequencies. In this study, willingness to pay was analyzed as a dichotomous outcome by calculating the proportion of participants who reported a positive willingness to pay, expressed as a percentage with confidence intervals. The factors associated with “willingness to pay” for solid waste management were analyzed using bivariate and multivariable logistic regression. At bivariate analysis, variables with a p-value <0.25 and had biological plausibility were adopted for multivariable analysis Multicollinearity was assessed using the Variance Inflation Factor (VIF). All independent variables had VIF values ranging from 1.12 to 2.34, indicating no evidence of problematic multicollinearity. Model specification was evaluated using the link test; the squared linear predictor was not statistically significant (p = 0.722), suggesting that the model was correctly specified and that no major specification errors were present. Model goodness-of-fit was further assessed using the Hosmer–Lemeshow test, which indicated an adequate fit between the observed and predicted outcomes (p = 0.604). Confounding was assessed using the 10% change in estimate rule by comparing the odds ratios across crude, partially adjusted and fully adjusted models. Age and sex were treated as a priori core covariates based on biological plausibility and consistent evidence from the literature indicating their potential confounding effects. Consequently, these variables were retained in the multivariable model regardless of their bivariate p-values, even when p < 0.25 was not met however, these variables met the multivariable inclusion. Other variables were considered for inclusion in the multivariable analysis if they met the p < 0.25 threshold at bivariate analysis. Factors with a P-value of <0.05 were considered significant at a 95% level of confidence.

### Ethical considerations

The study adhered to the ethical principles according to the Helsinki Declaration. It was reviewed and approved by the Research and Ethics Committee of Mbarara University of Science and Technology (MUST-2024–1781). Administrative clearance was obtained from the Mbarara District Chief Administrative Officer before data collection. Written informed consent was obtained from all participants as per standard human subject research practice. For individuals who could not read or write, the research assistants provided a detailed verbal explanation of the study procedures, risks, and benefits in the local language. After confirming understanding, these participants provided consent using their thumbprint. Participants’ identities were kept anonymous throughout the study. Privacy, confidentiality, and anonymity were ensured by conducting interviews in private settings and de-identifying all data. Identifiers were accessible only to the research team and solely for quality control purposes.

## Results

### Sociodemographic characteristics of the study participants

Of the 250 participants enrolled in this study, the majority, 61.2% (153/250), were female. The mean age of the study participants was 37.63 ± 11.8 years. More than half, 57.6% (144/250) of the participants were aged 18–38 years, and most, 80.4% (201/250) of the participants had some form of formal education. Regarding the amount of solid waste generated, the majority, 36% (90/250), produced 1–2 polythene bags of solid waste per week, and half, 51.6% (129/250), reported that solid waste was collected weekly from their households (Table 1).

**Table 1:** Sociodemographic characteristics of adult residents in households of Mbarara district, southwestern Uganda, March 2025.

Variable	Categories	Frequency (%)	Willingness to Pay	
			No n(%)	Yes n (%)
Sex	Female	153 (61.2)	65(42.5)	88(57.5)
	Male	97 (38.8)	29 (29.9)	68(70.1)
Age (in years) Mean ± SD = 37.6 ± 11.8	18-38 years	144 (57.6)	61(42.4)	83 (57.6)
	39-75 years	106 (42.4)	33 (31.1)	73 (68.9)
Education level	No formal education	51 (20.4)	22 (43.1)	29(56.9)
	Primary	102 (40.8)	39 (38.2)	63(61.8)
	Secondary	74 (29.6)	25 (33.8)	49(66.2)
	Tertiary	23 (9.2)	8 (34.8)	15(65.2)
Marital Status	Unmarried	49 (19.6)	21 (42.9)	28 (57.1)
	Married	201(80.4)	73(36.3)	128 (63.7)
House Ownership	Rent	173 (69.2)	72 (41.6)	101 (58.4)
	Self-owned	77 (30.8)	22 (28.6)	55 (71.4)
Monthly Income (Ugandan Shillings)	<100,000 UGX	93 (37.2)	46 (49.5)	47(50.5)
	≥100,000 UGX	157 (62.8)	48 (30.6)	109 (69.4)
The quantity of solid waste generated	Less than 1 Polythene bag	60 (24.0)	31 (51.7)	29 (48.3)
	1-2 Polythene bags	90 (36.0)	28 (31.1)	62 (68.9)
	2-8 Polythene bags	41 (16.4)	16 (39.0)	25 (61.0)
	More than 9 polythene bags	59 (23.6)	19 (32.2)	40 (67.8)
Places of waste disposal	Collected by private service providers	9 (3.6)	7 (77.8)	2 (22.2)
	Rubbish pit in the back yard	64 (25.6)	36 (56.2)	28 (43.8)
	Garbage dumping site	82 (32.8)	21 (25.6)	61 (74.4)
	Town Council Skip	95 (38.0)	30(31.6)	65 (68.4)
Frequency of collection of solid waste by service providers	Daily	89 (35.6)	38 (42.7)	51 (57.3)
	Weekly	129 (51.6)	40 (31.0)	89 (69.0)
	Monthly and not regularly collected	32 (12.8)	16 (50.0)	16 (50.0)

***Abbreviations:*** *SD, Standard Deviation; UGX, Ugandan Shillings; USD, United States Dollar.* **Note:** Currency conversion based on the exchange rate at the time of the study (approx. 1 USD ≈ 3,570 UGX). Thus, 100,000 UGX ≈ 28 USD

### Willingness to pay for solid waste management services

Out of the 250 participants enrolled in this study, 62% (156/250; 95% C.I.: 56.2%-68.2%) were willing to pay for solid waste management services. Of the 156 respondents who were willing to pay, the majority, 64.1% (100/156), were willing to pay about 0.30 USD (one thousand Uganda shillings), while a small proportion, 4.5% (7/156), were willing to pay less than 0.14 USD (five hundred Uganda Shillings) for solid waste management services.

Among the participants willing to pay for better solid waste management services, the most frequently cited reason that motivated participants to pay was keeping the environment clean and tidy, 59.0% (92/156), followed by health and disease prevention, reported 35.9% (56/156). Other notable reasons included conserving the environment, lack of nearby disposal grounds, and adherence to regulatory requirements.

### Factors associated with willingness to pay for solid waste management

The association of independent variables with willingness to pay for solid waste management services was investigated using both bivariate and multivariable logistic regression. At bivariate analysis, gender (cOR = 1.7, 95% CI: 1.0-3.0; *p*-value = 0.046), monthly income greater than 28 USD (cOR = 2.2, 95% CI: 1.3-3.8; *p*-value = 0.003), waste generation of 1–2 bags (cOR = 2.4, 95% CI: 1.2-4.7; *p*-value = 0.012), waste generation of more than 9 bags (cOR = 2.3, 95% CI: 1.1-4.9; *p*-value = 0.033), garbage dumping site as the place of waste disposal (cOR = 10.2, 95% CI: 2.0-52.8; *p*-value = 0.006), town council skip as the place of waste disposal (cOR = 7.6, 95% CI: 1.5 -38.7; *p*-value = 0.015), and weekly frequency of waste collection (cOR = 2.22, 95% CI: 1.01-4.89; *p*-value = 0.046) were significant.

In multivariable logistic regression, four factors remained statistically significant. Male participants had 2.4 higher odds of being willing to pay for solid waste management services (aOR = 2.4, 95% CI: 1.2-4.6; *p*-value = 0.011) compared to females. Participants with a monthly income greater than 28 USD had 2.2 times higher odds of being willing to pay (aOR = 2.2, 95% CI: 1.7-4.1; *p*-value = 0.015) compared to those with less than 28 USD. Participants who utilize town council services for solid waste disposal had 7.75 times higher odds of being willing to pay (aOR = 7.75, 95% CI: 1.35-44.47; *p*-value = 0.022) compared to those whose waste is collected by private service providers. However, this estimate should be interpreted with caution because the reference category (private service providers) had a small sample size (n = 9), which likely contributed to the wide confidence interval. Participants receiving weekly waste collection were significantly more willing to pay than those with monthly or irregular collection (aOR = 2.62; 95% CI: 1.06-6.50; *p* = 0.038) (Table 2).

**Table 2:** Bivariate and multivariable logistic regression of the factors associated with willingness to pay for waste management services among households in Mbarara district, southwestern Uganda, March 2025.

Variable	Bivariate analysis		Multivariable logistic regression analysis	
	cOR (95% CI)	P-Value	aOR (95% CI)	P-Value
**Age**				
18-38 Years	1		1	
39-75 Years	1.6 (1.0-2.8)	0.071*	1.2 (0.6-2.3)	0.575
**Gender**				
Female	1		1	
Male	1.7 (1.0-3.0)	0.046*	2.4 (1.2-4.6)	**0.011**
**Education level**				
No formal education	1			
Primary	0.8 (0.4-1.6)	0.560		
Secondary	1.2 (0.6-2.3)	0.545		
Tertiary	1.2 (0.5-3.0)	0.758		
**Marital Status**				
Unmarried	1			
Married	1.3 (0.8- 2.4)	0.398		
**Status of house ownership**				
Renting	1		1	
Owns the house	1.8 (1.0-3.2)	0.051*	1.6 (0.8 - 3.2)	0.148
**Monthly income**				
<100,000 UGX	1		1	
≥100,000 UGX	2.2 (1.3-3.8)	0.003*	2.2 (1.7-4.1)	**0.015**
**Solid waste generated**				
Less than 1 bag	1		1	
1-2 bags	2.4 (1.2-4.7)	0.012*	1.5 (0.7-3.5)	0.347
2-8 bags	1.7 (0.8-3.7)	0.212	1.1 (0.4-3.0)	0.911
More than 9 bags	2.3 (1.1-4.9)	0.033*	1.7 (0.6 - 4.4)	0.293
**Places of waste disposal**				
Collected by private service providers	1		1	
Rubbish pit in the backyard	2.7 (0.5-14.1)	0.233	1.9 (0.3-11.2)	0.478
Garbage Dumping Site	10.2 (2.0-52.8)	0.006*	5.7 (0.9-35.1)	0.061
Town Council Services	7.6 (1.5 -38.7)	0.015*	7.75 (1.35-44.47)	**0.022**
**Frequency of waste collection**				
Daily	1.34 (0.60-3.02)	0.477	1.34 (0.48-3.72)	0.570
Weekly	2.22 (1.01-4.89)	0.046*	2.62 (1.06-6.50)	**0.038**
Monthly and irregularly	1		1.00	

**Key:** *Variables included in multivariate analysis; ***Bold****: variables that remained significant at multivariate analysis at p < 0.05*.

## Discussion

Our findings indicate that 62% of participants were willing to pay for solid waste management services, a proportion that aligns closely with similar studies, such as 63% in Tanzania [19], 64.4% in Nigeria [17], 61% in Sri Lanka [28], and 64% in Kampala, Uganda [21]. This consistency suggests a general recognition across diverse settings of the importance of investing in waste management services. However, our observed willingness to pay is higher than the 48% reported in Lira City [20] and the 53.7% from a multi-city study in Ghana [29]. These differences may be context-specific and could be hypothesized to reflect variations in local economic conditions, household expenditure priorities, and perceptions of municipal service delivery. In semi-urban settings such as Mbarara, residents may place greater value on organized waste management services compared with larger urban centers. However, this interpretation remains speculative, as household economic indicators were not assessed in this study. In contrast, residents of larger urban centres may experience higher financial burdens, which could limit their ability or willingness to pay for such services [30]. On the other hand, our findings fall below the 83% reported in Nepal [31] and >86% in Ethiopia [32,33], where studies employed the contingent valuation method, an approach known to prompt more favourable responses toward payment. This methodological difference could partly explain the higher willingness observed in those contexts. Nonetheless, the 62% WTP identified in our study demonstrates a substantial level of public support for structured waste management systems in Mbarara District. It presents a valuable opportunity for local governments and private stakeholders to design and implement sustainable, cost-recovery models for SWM services. Additionally, with 64.1% of participants willing to pay 0.30 USD (approximately UGX 1000) or more for these services, there is potential to introduce affordable service fees. However, any payment model must remain sensitive to the socio-economic realities of the population to ensure equity, accessibility, and long-term sustainability. Challenges in SWM, including financial constraints and the need for effective service models, are similarly documented in other low-resource settings, such as in healthcare facilities in Nepal [34].

We found that male participants had 2.4 higher odds of being willing to pay for solid waste management services compared to their female counterparts. Our finding is consistent with those obtained in studies done in Ethiopia [35] and China [36]. On the contrary, previous studies reported female gender as being associated with willingness to pay for solid waste management services [32,37], suggesting that gender differences in willingness to pay may be highly context-specific.

Additionally, the proportion of men earning more than 28 USD a month was 69.1% (67/97) higher than that of women at 58.8% (90/153 which may partly explain the observed gender differences. In this context, men may be more likely to express willingness to pay due to their relatively greater access to income and their role in household financial decision-making, as documented in similar settings. At the same time, socio-cultural roles may also influence this pattern: women are often responsible for household waste management and may prioritize immediate household needs over additional service payments, particularly under financial constraints [38]. These interpretations should be understood within the local socio-economic and cultural context and not generalized across settings. Furthermore, men might have a different perception of the socioeconomic benefits associated with improved SWM as previously reported in literature [39]. Since men are the majority heads of households, this finding is key in guiding the design and implementation of sustainable Solid waste management strategies in Mbarara District. Income and gender differences in willingness to pay highlight equity concerns, as women and lower-income households may have limited financial capacity, requiring targeted strategies to ensure fair access to solid waste management services.

Participants with a monthly income greater than 28 USD had 2.2 higher odds of being willing to pay compared to those with less than 28 USD. This is consistent with previous findings where increased income is a significant factor [20,28,31,33,37]. In the context of waste management, households with high income may value services that prevent potential health risks and improve community cleanliness [39]. This highlights an opportunity for local governments and private providers to mobilize financial contributions more effectively from middle- and higher-income segments, who are both willing and able to pay. As such, it is important to tailor fee structures to reflect these income differences to ensure affordability and maximize revenue generation for sustainable service provision.

We found that participants who disposed of their waste through town council services had 7.75 higher odds of willing to pay for solid waste management services compared to those whose waste was collected by private providers. This finding is consistent with previous research suggesting that public waste services are often more widespread and perceived as more legitimate or community-oriented, fostering a stronger sense of shared responsibility and public trust [14,40,41]. Similar challenges and practices in Nepal highlight the central role of municipal government and the importance of community engagement in effective SWM [34,41]. Town council services may also be more accessible to a wider socioeconomic spectrum, particularly in semi-urban settings where private waste management options are limited or more expensive. In contrast, the use of private providers is frequently observed in areas underserved by public systems or among higher-income households who can afford individualized services [42]. This disparity in WTP based on service provider highlights the importance of developing an inclusive and equitable SWM strategy, one that ensures both accessibility and affordability across diverse communities. Strengthening public waste services, alongside regulated collaboration with private actors, may enhance public willingness to financially support sustainable waste management systems.

Study findings show a positive association between the frequency of garbage collection and the participants’ willingness to pay for SWM services. Participants receiving weekly waste collection were almost three times more willing to pay than those whose waste was collected monthly or irregularly. This relationship indicates that the perceived value and reliability of waste management services greatly influence household decisions to provide financial support. Regular and predictable collection not only reduces the health and environmental hazards associated with waste accumulation but also builds public trust and satisfaction with the service, thereby increasing the likelihood of cost-sharing participation [37,43]. The higher WTP for this consistent service shows that residents are likely willing to contribute more financially when they experience tangible and regular benefits from SWM services. As such, local authorities and private waste managers should prioritize regular service delivery as a strategic lever to foster greater willingness among residents to invest in sustainable SWM systems.

### Study strengths and limitations

This study provides important evidence on the factors associated with willingness to pay for solid waste management in Mbarara District, southwestern Uganda. However, this study has some limitations that need to be accounted for in the interpretation of its findings. The study has several methodological limitations. First, the use of consecutive household sampling may have introduced potential selection bias, as households that were more accessible were more likely to be included. Second, interviewer effects cannot be fully ruled out, and participants may have provided responses they perceived as socially acceptable, introducing social desirability bias, especially for self-reported practices and willingness to pay. Third, we did not measure certain contextual variables, such as proximity to waste skips, prior exposure to enforcement activities, or other environmental factors that may confound the observed associations. Furthermore, our measurement approach for WTP did not employ contingent valuation or discrete choice methods; instead, WTP was assessed dichotomously, which limits our ability to estimate actual price points and may influence the interpretation of economic. In addition, the small sample in the private service provider category (n = 9) contributed to wider confidence intervals in the logistic regression estimates. While category consolidation was considered, it was not implemented to preserve the distinct behavioural differences observed between users of private versus public services.

This study employed a clustered sampling approach at the village level; however, adjustment for clustering was not incorporated during sample size estimation or statistical analysis. Village-level identifiers were not collected, which limited our ability to apply cluster-robust standard errors or survey weighting procedures. As a result, analyses were conducted using unweighted data. This may have affected variance estimation, potentially leading to underestimated standard errors and confidence intervals that are narrower than would be expected under a fully weighted or cluster-adjusted analysis.. Future studies should ensure the collection of cluster identifiers to allow appropriate adjustment for clustering at both the design and analysis stages.

Future research should build on these findings by employing WTP preference methods such as discrete choice experiments to estimate actual payment thresholds and identify which service attributes, for example, frequency, distance to collection points, provider type, residents value most. Such evidence would provide policymakers with precise, actionable insights for designing cost-effective, responsive, and sustainable waste management services aligned with community preferences findings.

### Public health and policy implications

This study underscores the need for inclusive and equitable financing models for SWM. Male headed and higher-income households were more willing to pay for solid waste management services, reflecting disparities in financial capacity and intra-household decision-making. To address these inequities, gender responsive strategies should be integrated into SWM programs in Mbarara District. Engagement and sensitization efforts should deliberately target women who are primary household waste handlers through women’s groups, savings groups, markets, and village health teams. Flexible payment options, such as small installment payments, mobile money, or group based schemes, may reduce financial and decision-making barriers for women. Involving women in community waste management committees can further strengthen their participation and support equitable, sustainable SWM implementation.

Policymakers should consider tiered or subsidized payment structures that account for income differences to enhance affordability while maximizing revenue generation for sustainable service provision. Operationally, tiered fees could be set based on household income categories, with low-income or vulnerable households identified through local council records or community verification receiving reduced tariffs or partial subsidies, delivered through existing town council billing systems or mobile money payment platforms. Moreover, the higher willingness to pay among residents using public town council services suggests that strengthening public waste services through reliable collection schedules and timely response to service complaints can increase community participation and trust. Designing cost recovery models that balance accessibility, equity, and financial sustainability will be critical for long-term success. Furthermore, ensuring transparency in the use of collected fees and maintaining consistent service delivery will further support cost recovery models that balance accessibility, equity, and long-term financial sustainability.

Finally, the study provides actionable insights for behavior-focused interventions. Public awareness campaigns that emphasize the tangible health and environmental benefits of reliable SWM, alongside community engagement strategies, could improve service uptake and promote timely payment for waste services, particularly among women and lower-income households who were less willing to pay. Policymakers can use these findings to design targeted interventions that strengthen participation, improve payment compliance, and mobilize both public and private resources to support sustainable and equitable waste management systems in Mbarara District and similar semi-urban settings.

## Conclusion

Nearly two-thirds of the participants were willing to pay for solid waste management services in this study. Willingness to pay was associated with male gender, monthly income above 28 USD, reliance on town council waste collection services and weekly collection of the solid wastes. The town council leadership needs to prioritize regular investment in solid waste management services to ensure that waste collection and disposal occur at least weekly. Furthermore, the councils should establish an inclusive and flexible payment structure that considers the diverse income levels and payment preferences of town dwellers, as these factors significantly influence willingness to pay. Future studies should apply WTP preference methods, such as discrete choice experiments, to determine actual payment thresholds and the service attributes most valued by residents. This will generate clearer evidence to guide the design of cost-effective and sustainable waste management services that align with community preferences.

## Supporting information

S1 Data Data set. (XLS)

S1 Fig Map of Mbarara District. (DOCX)

S1 Text Data collection tool. (DOCX)

## Abbreviations

WTP: Willingness to pay
SWM: Solid waste management
UGX: Ugandan shillings
MUST: Mbarara University of Science and Technology
cOR: crude odds ratio
aOR: adjusted odds ratio

## References

[1] Chen DM-C, Bodirsky BL, Krueger T, Mishra A, Popp A. The world’s growing municipal solid waste: trends and impacts. Environ Res Lett. 2020;15(7):074021. doi: 10.1088/1748-9326/ab8659

[2] Hoornweg D, Bhada-Tata P. What a waste: a global review of solid waste management. 2012.

[3] UNEP. Global waste management outlook 2024; 2024. Available from: https://www.unep.org/resources/global-waste-management-outlook-2024

[4] Gedefaw M. Assessing the current status of solid waste management of Gondar town, Ethiopia. Int J Sci Technol Res. 2015;4(9):28–36.

[5] Medina M. Solid wastes, poverty and the environment in developing country cities: challenges and opportunities: WIDER Working Paper; 2010.

[6] Adedara ML, Taiwo R, Bork H-R, editors. Municipal solid waste collection and coverage rates in Sub-Saharan African countries: a comprehensive systematic review and meta-analysis. In: Waste. MDPI; 2023.

[7] Scarlat N, Motola V, Dallemand JF, Monforti-Ferrario F, Mofor L. Evaluation of energy potential of municipal solid waste from African urban areas. Renew Sustain Energy Rev. 2015;50:1269–86. doi: 10.1016/j.rser.2015.05.067

[8] Okot-Okumu J, Nyenje R. Municipal solid waste management under decentralisation in Uganda. Habitat Int. 2011;35(4):537–43. doi: 10.1016/j.habitatint.2011.03.003

[9] UBOS. Uganda Bureau of Statistics 2017 statistical abstract. Uganda Bureau of Statistics; 2017. p. 1–341.

[10] Mesharch WK, Boaz N, Derrick K. The neglected governance challenges of solid waste management in Uganda: insights from a newly created city of Mbarara. 2022.

[11] Gilbert N, Ziqiang Y, Hongzhi M. Current situation of solid waste management in East African countries and the proposal for sustainable management. Afr J Environ Sci Technol. 2021;15(1):1–15. doi: 10.5897/ajest2020.2911

[12] Mukama T, Ndejjo R, Musoke D, Musinguzi G, Halage AA, Carpenter DO, et al. Practices, concerns, and willingness to participate in solid waste management in two urban slums in Central Uganda. J Environ Public Health. 2016;2016:6830163. doi: 10.1155/2016/6830163 27066081 PMC4808673

[13] Ssemugabo C, Wafula ST, Lubega GB, Ndejjo R, Osuret J, Halage AA, et al. Status of household solid waste management and associated factors in a slum community in Kampala, Uganda. J Environ Public Health. 2020;2020:6807630. doi: 10.1155/2020/6807630 32454842 PMC7225857

[14] Katusiimeh MW. Public and private service provision of solid waste management in Kampala, Uganda; 2012.

[15] Bah YM, Artaria MD. Privatization of solid waste management: opportunities and challenges. Indones J Urban Environ Technol. 2021;4(2):142–63. doi: 10.25105/urbanenvirotech.v4i2.8219

[16] Gumisiriza P, Kugonza S. Corruption and solid waste management in Mbarara Municipality, Uganda. J Environ Public Health. 2020;2020:4754780. doi: 10.1155/2020/4754780 32676123 PMC7341428

[17] Oyawole FP, Ajayi OP, Aminu RO, Akerele D. Willingness to pay for improved solid waste management services in an urbanizing area in South-East Nigeria. EJESM. 2016;9(6):793–803. doi: 10.4314/ejesm.v9i6.11

[18] Batu MM, Tolosa EF. Determinants of households’ willingness to pay for improved solid waste management in Ethiopia: the case study of Jimma Town. J Environ Earth Sci. 2016;6(2224–3216):75.

[19] Mussa J. Residents’ willingness to pay for improved solid waste management in Dodoma municipality, Tanzania. Sokoine University of Agriculture; 2015.

[20] Apio E, Opio B, Acanga A, Akello AR. Factors influencing willingness to pay for improved solid waste collection services among households in urban cities in Uganda: empirical evidence from Lira City. BMC Public Health. 2024;24(1):2150. doi: 10.1186/s12889-024-19568-6 39112956 PMC11308488

[21] James M, Nabavubya NF. Assessment of willingness to pay for solid waste collection services in Uganda: a case study of Kawempe Division. Available at SSRN 3177076; 2017.

[22] Yasin AS. Assessing households’ willingness to pay for improved solid waste management services in Jigjiga, Ethiopia. EER. 2021;9(2):39–44. doi: 10.13189/eer.2021.090201

[23] Opoku L, Addai B, Gyimah AG, Asante F. Household willingness to pay for waste management service. Rural Soc. 2024;33(1):52–64. doi: 10.1080/10371656.2023.2301196

[24] Nahamya J. Mbarara municipality: locals protest poor services, want mayor to step down. Kampala, Uganda: Soft Power Uganda; 2019.

[25] Kushaba A. Mbarara traders, municipal bicker over garbage management. Kampala, Uganda: Uganda Radio Network; 2017.

[26] Kabasongora M. UGX 556 million Mbarara garbage recycling project stalls. Kampala; 2011.

[27] Mukombozi R. Garbage chokes Mbarara town as residents close site, Daily Monitor. Kampala, Uganda; 2011.

[28] Dilsath A, Prasada DVP. Assessing the potential for an improved solid waste collection in Kalmunai, Sri Lanka: an analysis of willingness to pay. Trop Agric Res. 2021;32(4):434. doi: 10.4038/tar.v32i4.8512

[29] Boateng KS, Agyei-Baffour P, Boateng D, Rockson GNK, Mensah KA, Edusei AK. Household willingness-to-pay for improved solid waste management services in four major Metropolitan cities in Ghana. J Environ Public Health. 2019;2019:5468381. doi: 10.1155/2019/5468381 30719051 PMC6334316

[30] Adabor O, Mintah K, Amankwah E. The effect of financial inclusion on urban population in Ghana. Soc Sci Quart. 2023;104(4):742–60. doi: 10.1111/ssqu.13267

[31] Bhattarai K. Households’ willingness to pay for improved solid waste management in Banepa municipality, Nepal. Environ Nat Resour J. 2015;13(2):14–25.

[32] Tassie K, Endalew B. Willingness to pay for improved solid waste management services and associated factors among urban households: One and one half bounded contingent valuation study in Bahir Dar city, Ethiopia. Cogent Environ Sci. 2020;6(1). doi: 10.1080/23311843.2020.1807275

[33] Kaso AW, Hareru HE, Ashuro Z, Soboksa NE. Assessment of households’ willingness to join and pay for improving waste management practices in Gedeo zone, southern Ethiopia. BioMed Res Int. 2022;2022(1):9904665. 36164445 10.1155/2022/9904665 PMC9509238

[34] Aryal M, Adhikary S, Aryal V, Adhikari R. An analysis of solid waste management practices in Lamjung District Community Hospital, Nepal. J Wastes Biomass Manag. 2019;1(2):09–13. doi: 10.26480/jwbm.02.2019.09.13

[35] Mulat S, Worku W, Minyihun A. Willingness to pay for improved solid waste management and associated factors among households in Injibara town, Northwest Ethiopia. BMC Res Notes. 2019;12(1):401. doi: 10.1186/s13104-019-4433-7 31300047 PMC6626345

[36] Trang PTT, Toan DQ, Hanh NTX, editors. Estimating household willingness to pay for improved solid waste management: a case study of Thu Dau Mot city, Binh Duong. MATEC Web of Conferences. EDP Sciences; 2017.

[37] Chernet D, Sema W, Gebeyehu A, Wogayehu BT. Willingness to pay for improved solid waste management and associated factors among households in Debre Berhan town, North Shoa Zone, Amhara, Ethiopia, 2022. Front Sustain. 2024;5:1463777.

[38] Amoah JO, Britwum AO, Essaw DW, Mensah J. Solid waste management and gender dynamics: evidence from rural Ghana. Res Glob. 2023;6:100111. doi: 10.1016/j.resglo.2023.100111

[39] Uma KE, Nwaka ID, Nwogu MU, Obidike PC. What are the triggers of household decision-making on waste disposal choices? A gender differentiated analysis. Heliyon. 2020;6(12):e05588. doi: 10.1016/j.heliyon.2020.e05588 33319089 PMC7726665

[40] Awunyo-Vitor D, Ishak S, Seidu Jasaw G. Urban households’ willingness to pay for improved solid waste disposal services in Kumasi Metropolis, Ghana. Urban Stud Res. 2013;2013:1–8. doi: 10.1155/2013/659425

[41] Aryal P, Adhikari P. Investigating household knowledge, attitudes, and practices in solid waste segregation, recycling and management: a study of Kathmandu Metropolitan City. MVIC J Mgt Info Technol. 2025;1(1):29–41. doi: 10.3126/mvicjmit.v1i1.77315

[42] Katusiimeh MW, Mol APJ, Burger K. The operations and effectiveness of public and private provision of solid waste collection services in Kampala. Habitat Int. 2012;36(2):247–52. doi: 10.1016/j.habitatint.2011.10.002

[43] Sujauddin M, Huda SMS, Hoque ATMR. Household solid waste characteristics and management in Chittagong, Bangladesh. Waste Manag. 2008;28(9):1688–95. doi: 10.1016/j.wasman.2007.06.013 17845843

