# Supplementary figures and images for: Willingness to pay for solid waste management services and associated factors in Mbarara District, Southwestern Uganda
Source: PLOS Glob Public Health. 2026 Mar 26;6(3):e0005175. doi: 10.1371/journal.pgph.0005175 (PMC13020830; doi:10.1371/journal.pgph.0005175)

**Fig 1: Showing the map of Mbarara District**


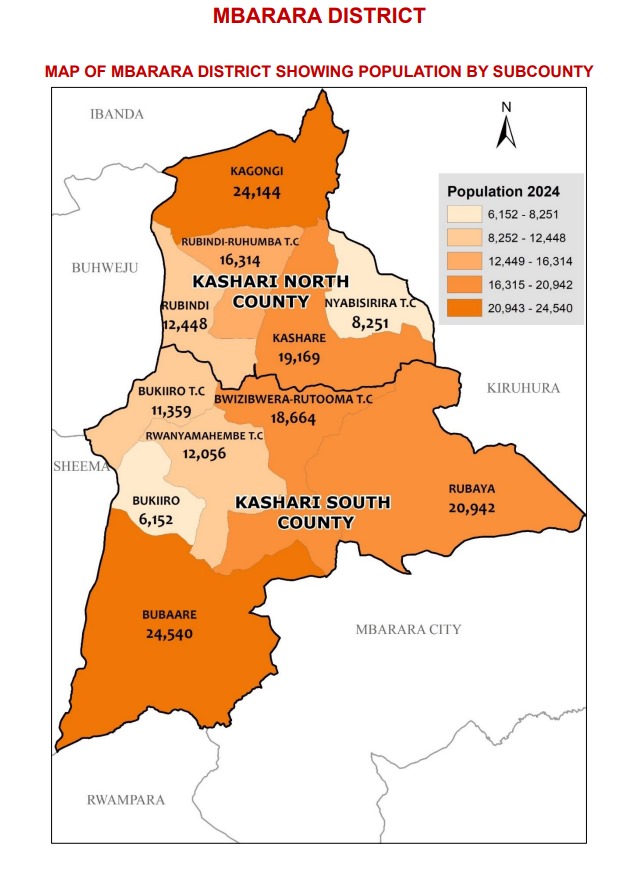

Supplement: S1 Fig — (DOCX) [file pgph.0005175.s002.docx]
