# Supplementary material for: Willingness to pay for solid waste management services and associated factors in Mbarara District, Southwestern Uganda
Source: PLOS Glob Public Health. 2026 Mar 26;6(3):e0005175. doi: 10.1371/journal.pgph.0005175 (PMC13020830; doi:10.1371/journal.pgph.0005175)
Supplement: S1 Text — (DOCX) [file pgph.0005175.s003.docx]

# **Data Collection tool**

**Section a: Social demographic, social economic and household Factors affecting the WTP for waste management services in Mbarara district.**

1. Gender: Male Female

2. Age of the Respondent

| S/n | Age bracket |  |
| --- | --- | --- |
| 1 | 15-19years |  |
| 2 | 20-34 years |  |
| 3 | 35-49years |  |
| 4 | 50 years and Above |  |

3. Educational attainment of the respondent

| S/n | Education Level |  |
| --- | --- | --- |
| 1 | Pre-primary |  |
| 2 | PLE |  |
| 3 | UCE |  |
| 4 | UACE |  |
| 5 | Certificate/ Diploma |  |
| 6 | Bachelors / Masters |  |

4. Marital status of the Respondent

| S/n | Marital status |  |
| --- | --- | --- |
| 1 | Single |  |
| 2 | Married |  |
| 3 | Widowed |  |
| 4 | Separated |  |

5. House ownership

| S/n | House ownership |  |
| --- | --- | --- |
| 1 | Self-owned |  |
| 2 | Rented |  |
| 3 | Institution |  |
| 4 | Friend/ Relative |  |

1. Monthly Income levels:

| S/n | Monthly income |  |
| --- | --- | --- |
| 1 | Less than 100,000 |  |
| 2 | 100,000-490,000 |  |
| 3 | 500,0000-990,000 |  |
| 4 | 1,000,000 and Above |  |

1. Do you generate waste at the household level Yes NO
2. List the types of wastes generated……………………………………………………… …………………………………………………………………………………………...…………………………………………………………………………………………
3. How much solid waste does it generate per week? Estimate using the amount that would fill a polythene bag.

| S/n | Amount of waste generated |  |
| --- | --- | --- |
| 1 | Less than a polythene bag |  |
| 2 | 1-2 polythene bags |  |
| 3 | 3-4 polythene bags |  |
| 4 | 5-6 polythene bags |  |
| 5 | 7-8 polythene bags |  |
| 6 | 9 and Above |  |
| 7 | I Don’t Know |  |

1. Where is the waste generated disposed off?

- Town Council Skip
- Rubbish pit in the Backyard
- Garbage Dumping site
- Other (specify)……………………………

**For those whose waste is collected by service providers;**

1. Which company collects and manages your waste?

…………………………………………….

1. How much are you charged by that company?

| S/n | Amount Category |  |
| --- | --- | --- |
| 1 | For free |  |
| 2 | Between UGx 500- 1,000 |  |
| 3 | Above UGx 1,000 |  |

1. How often is the Waste generated collected?

| S/n | Period of collection |  |
| --- | --- | --- |
| a. | Daily |  |
| b. | Weekly |  |
| c. | Monthly |  |
| d. | Not Regularly collected |  |

1. Who pays for the management of solid waste services in the Town Council?

-Town Council

-NGO

-Households/User fees

If you already pay for the management of solid waste, go to section C questions. For those who do not pay for the management of solid waste, **answer section B**.

**Section B: Assessing the WTP for Management of Solid Waste by Households**

1. Are you willing to pay for the management of Solid waste services in this Town?

| S/N | Answer | Options |
| --- | --- | --- |
| 1 | Willing to pay |  |
| 2 | Not Willing to pay |  |

1. What amount are you prepared to offer as waste management fees?

| S/n | Amount Category |  |
| --- | --- | --- |
| 1 | Less than UGx 500 |  |
| 2 | Between UGx 500- 1,000 |  |
| 3 | Above UGx 1,000 |  |

1. How often would you wish your waste to be collected?

| S/n | Days category |  |
| --- | --- | --- |
| 1 | Daily |  |
| 2 | Two days per week |  |
| 3 | Weekly |  |
| 4 | Another period (specify) |  |

1. What are the reasons that hinder you from paying for better management services for solid waste?

**……………………………………………………………………………………………………………………………………………………………………………………………………………………………………………………………………………….**

**If you are already paying for waste management, answer section c**

**Section c**

1. Are you willing to continue paying for the management of Solid waste services in this Town?

| S/N | Answer | Options |
| --- | --- | --- |
| 1 | Willing to pay |  |
| 2 | Not Willing to pay |  |

1. What amount are you prepared to offer as waste management fees?

| S/n | Amount Category |  |
| --- | --- | --- |
| 1 | Less than UGx 500 |  |
| 2 | Between UGx 500- 1,000 |  |
| 3 | Above UGx 1,000 |  |

1. How often would you wish your waste to be collected?

| S/n | Days category |  |
| --- | --- | --- |
| 1 | Daily |  |
| 2 | Two days per week |  |
| 3 | Weekly |  |
| 4 | Another period (specify) |  |

1. What are the reasons that motivate you to pay for better management services for solid waste

**……………………………………………………………………………………………………………………………………………………………………………………**

**Section D: Knowledge. Attitudes And Practices That impact on the WTP For the management of solid waste In Mbarara District**

Please indicate your level of agreement or disagreement with each statement below by selecting the appropriate box on the Likert scale provided.

| Strongly Concur (SC) | Concur (C) | Unsure ( U) | Dispute (D) | Strongly Dispute (SD) |
| --- | --- | --- | --- | --- |
| 5 | 4 | 3 | 2 | 1 |

| No | **Assessing the generation of wastes** | SC | C | U | D | SD |
| --- | --- | --- | --- | --- | --- | --- |
| 1 | Households generate the majority of solid waste compared to other sources. |  |  |  |  |  |
| 2 | The garbage I produce is stored in a dustbin or Another specially designed container. |  |  |  |  |  |
| 3 | I typically keep the solid waste I produce in a” kadeya: or bags. |  |  |  |  |  |
| 4 | I consistently divide biodegradable and non-biodegradable waste, storing them in separate containers. |  |  |  |  |  |
| 5 | I actively reduce the amount of solid waste that I generate at the households/institutions where I work. |  |  |  |  |  |
| 6 | The solid waste I produce consists of either food waste, paper, yard trimmings, plastics, glass, metals, or wood. |  |  |  |  |  |
|  | **Statements measuring Waste Handling** |  |  |  |  |  |
| 1 | The town council collects the solid waste that I produce from my premises or in the neighbourhood. |  |  |  |  |  |
| 2 | I frequently use the private waste collector for collection of the solid waste I produce at my household. |  |  |  |  |  |
| 3 | I transport my accumulated solid waste to a designated dumping site. |  |  |  |  |  |
| 4 | The solid waste I gather should undergo treatment at an authorized sanitary landfill. |  |  |  |  |  |
| 5 | We possess adequately trained personnel to manage the treatment of solid waste. |  |  |  |  |  |
| 6 | I advocate for the implementation of the reduce, recycle, reuse approach to achieve efficient solid waste management. |  |  |  |  |  |
|  | **Statements measuring waste generator** |  |  |  |  |  |
| 1 | Households are part of the solid waste generators in my area. |  |  |  |  |  |
| 2 | Markets are part of solid waste producers in my area. |  |  |  |  |  |
| 3 | Commercial premises are producers of solid waste in my area. |  |  |  |  |  |
| 4 | Agricultural activities produce solid waste in my area. |  |  |  |  |  |
| 5 | Slaughterhouses contribute to producing solid waste in my area. |  |  |  |  |  |
| 6 | Healthcare facilities produce solid waste in my area. |  |  |  |  |  |
| 7 | Informal sector industries are producers of solid waste in my area. |  |  |  |  |  |

**Section E: Knowledge of the impact of poor solid waste management on public health**

1. Are you aware that improper waste management can affect public health?
2. Yes
3. No
4. Which of the following health problems do you think are associated with poor solid waste management? (Select all that apply)
5. Diarrhea
6. Respiratory diseases
7. Vector-borne diseases (e.g., malaria, dengue)
8. Skin infections
9. Other (please specify)
10. I don’t know
11. In your opinion, what are the main health risks posed by poorly disposed solid waste in your community?
12. Water contamination
13. Spread of disease
14. Air pollution
15. Breeding of pests (e.g., rats, flies)
16. Other (please specify)
17. Do you think improper waste disposal can lead to an outbreak of diseases in your community?
18. Yes
19. No
20. Unsure
